# Clocks Underneath: The Role of Peripheral Clocks in the Timing of Female Reproductive Physiology

**DOI:** 10.3389/fendo.2013.00091

**Published:** 2013-07-23

**Authors:** Michael T. Sellix

**Affiliations:** ^1^Department of Medicine, Division of Endocrinology and Metabolism, School of Medicine and Dentistry, University of Rochester, Rochester, NY, USA

**Keywords:** clock gene, reproduction, fertility, circadian rhythm, ovary, uterus, oviduct, pituitary gland

## Abstract

The central circadian pacemaker in the suprachiasmatic nucleus (SCN) is a critical component of the neuroendocrine circuit controlling gonadotropin secretion from the pituitary gland. The SCN conveys photic information to hypothalamic targets including the gonadotropin releasing hormone neurons. Many of these target cells are also cell autonomous clocks. It has been suggested that, rather then being singularly driven by the SCN, the timing of gonadotropin secretion depends on the activity of multiple hypothalamic oscillators. While this view provides a novel twist to an old story, it does little to diminish the central role of rhythmic hypothalamic output in this system. It is now clear that the pituitary, ovary, uterus, and oviduct have functional molecular clocks. Evidence supports the notion that the clocks in these tissues contribute to the timing of events in reproductive physiology. The aim of this review is to highlight the current evidence for molecular clock function in the peripheral components of the female hypothalamo-pituitary-gonadal axis as it relates to the timing of gonadotropin secretion, ovulation, and parturition.

## Introduction

Generating a complete picture of the timing systems role in pregnancy and parturition requires examining the molecular clocks role in the timing of events that precede fertilization. The temporal control of ovulation and the events that follow depend in large part on the timing of luteinizing hormone (LH) and follicle stimulating hormone (FSH) secretion from the pituitary gland (1). Serum gonadotropin levels display robust diurnal variation (2–4). In nocturnal rodents, LH secretion increases in the afternoon and peaks 3–4 h into the night (3, 5). These rhythms are dependent upon the activity of pacemaker neurons in the suprachiasmatic nucleus (SCN) (6, 7). As with behavior, gonadotropin secretory rhythms persist in constant conditions (8, 9). Neuropeptidergic SCN efferents pass temporal cues from the retina to gonadotropin releasing hormone (GnRH) neurons in the preoptic area of the basal forebrain (10, 11). GnRH stimulates gonadotropin secretion from the pituitary gland via the portal vasculature. The timing of the ovulation-inducing surge of LH depends on this neuroendocrine network (12, 13). It has long been the view that this complex circuit is the sole source for timing cues in the female reproductive system (5, 14).

**Figure 1:**
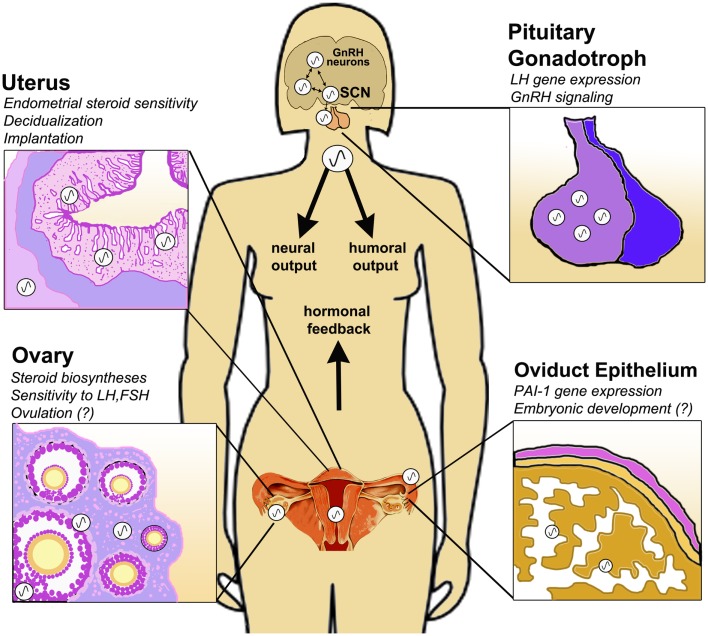
**Circadian clock function in the peripheral tissues of the female HPG axis**. The central circadian clock in the suprachiasmatic nucleus (SCN) drives rhythmic GnRH secretion and subsequent gonadotropin secretion from the pituitary. In addition to these neuroendocrine pacemakers, clocks are also present in the pituitary gonadotroph, uterine endometrium and myometrium, oviduct epithelial cells and ovarian theca, interstitial, and granulosa cells. Clock function has been implicated in GnRH signaling, gonadotropin sensitivity, ovulation, steroid hormone synthesis, embryonic maturation, implantation, and decidualization. Synchronization of central and peripheral oscillators is mediated by several putative humoral and neural cues, driven either directly or indirectly by the SCN. Moreover, feedback signals from the periphery, e.g., steroid hormones of ovarian origin, modulate the timing of the clock in both central and peripheral tissues of the HPG axis.

The biochemical substrate for circadian oscillations is a transcription-based autoregulatory negative feedback loop of interacting clock gene transcription factors, including at its core the transcriptional enhancer *bmal1* and the repressors *period (1,2)* and *cryptochrome (1,2)* (15). In addition to SCN neurons, the pituitary, ovary, uterus, and oviduct are each comprised of cell-autonomous circadian clocks [see Figure 1; (16–23)]. However, a functional role for the clock in these tissues, particularly with regard to the timing of ovulation, implantation, and parturition, has yet to be thoroughly defined (24, 25). The clock in the ovary may play a significant role in the timing of ovulation, steroid hormone synthesis, follicular growth, and differentiation (26–30). Clock genes in the uterus and oviduct have been implicated in the processes of implantation, embryo maturation, development of the fetus, and eventual parturition (18, 23, 31–33). Others have linked circadian clock function to reproductive physiology, with particular emphasis on steroid hormone biosynthesis (34–36). Mutations altering clock gene expression have a substantial impact on reproductive function in both rodents (31, 33, 37, 38) and humans (39).

Taken together, these data indicate that while oscillators in the basal hypothalamus play a critical role, the peripheral components of the hypothalamo-pituitary-gonadal (HPG) axis may also contribute to the timing of reproductive physiology. Disruption of the molecular clock in these peripheral tissues or reduced synchrony amongst these oscillators may be a factor in diseases that cause infertility (40). The goals of this review are: (1) highlight the evidence for molecular clock function in the peripheral tissues of the HPG axis and (2) briefly speculate on the physiological ramifications of disrupted molecular clock function as it relates to ovulation and the events that follow. For the purpose of this mini-review, we will avoid discussion of the complex and well-described role of the clock genes in photoperiod-dependent reproductive physiology. Our intention is to shed light on the most salient and current evidence for peripheral clock function in basic female reproductive physiology and highlight potential impacts of circadian disruption on fertility.

## Circadian Clock Form and Function: The Pituitary Gonadotroph

Both circadian and ultradian patterns of LH secretion have been described in female mammals (41–45). Examination of LH release from isolated pituitary explants and pituitary cell cultures indicated that individual gonadotrophs or a subpopulation of differentially regulated gonadotrophs may be autonomous circadian oscillators (44, 46). More recently several groups have described cell autonomous clock gene expression in the pituitary gland (16, 22, 47–52). However, the evidence for molecular clock function in specific hormone secreting cells is limited to gonadotrophs and lactotrophs (22, 50, 51). Kakar and colleagues provided the earliest evidence for a functional clock in gonadotrophs with the revelation that GnRH induces *per1* expression in gonadotroph cell lines (53). Olcese and colleagues subsequently determined that *per1*, but not *per2*, gene expression was activated by GnRH receptor (GnRHR) through MAP kinase-dependent signaling (54). This group also identified seven clock-gene target sequences in the mouse GnRHR promoter and determined that both BMAL1 and CLOCK bind to and activate GnRHR expression (22). Most importantly they were able to co-localize PER1 with LH in pituitary cells *in situ* (22). Further, using siRNA they confirmed that suppression of *bmal1* expression effectively reduced GnRHR mRNA. Finally, this group reported that GnRH mediated activation of early growth response protein-1 (EGR-1) also leads to activation of *per1* expression (51).

In addition to GnRH signaling and receptor gene expression, the molecular clock may also regulate physical changes in the pituitary. That is, gonadotroph proliferation changes during the estrous cycle (55) and exhibits a diurnal rhythm, with a peak in S-phase near 14:00 h (56). A rhythm of gonadotroph proliferation with a period equal to the 4-day estrous cycle and a peak on estrous was described in rats (57). Together, these data indicate that the circadian clock in gonadotrophs may regulate rhythms of cell proliferation, secretory responses to gonadotropins, and gonadotropin gene expression. Recently, it was reported that only *per1* mRNA was rhythmically expressed in human pituitary glands (58). Surprisingly, a rhythm of PER1 protein was not detected.

Finally, it was recently reported that, unlike global *bmal1* deletion, cell-specific deletion of *bmal1* in gonadotrophs had no effect on the amplitude and timing of gonadotropin secretion (52). Mice with gonadotroph-specific *bmal1* KO had normal fertility, though the duration of the estrous cycle was increased (52). These data suggest that molecular clock function in regions upstream (basal hypothalamus) or downstream (ovary) may be more critical for normal reproductive function in mice.

## Circadian Clock Form and Function: The Ovarian Follicle

Rhythmic expression of clock genes in the ovary has been observed in rat (19–21, 26), mouse (59), quail (60), and chicken (61). In 2006 a pair of independent studies reported rhythms of clock gene expression in the rat ovary (19, 20). These studies revealed that gonadotrophin exposure *in vivo* induced cyclic expression of *bmal1* and *per2* mRNA in the ovaries of hypophysectomized prepubertal rats (20). They also observed diurnal rhythms of *per1* and *per2* expression that persisted across the reproductive cycle (19). Further, these authors confirmed rhythms of clock gene expression within large preantral follicles, small antral follicles, graafian follicles, and corpora lutea. The same group have subsequently confirmed this finding (29). More recently it was reported that rhythms of clock gene expression are only present in mature isolated granulosa and luteal cells (21, 26, 62, 63). Comparable results have also been reported in quail, with only the largest preovulatory follicles showing rhythmic *per2* expression (60). The absence of rhythmic clock gene expression in immature or differentiating cells has also been reported in the thymus and testis (34). These data suggest that circadian rhythms of clock gene mRNA are “activated” at some point during differentiation of follicular cells. Studies on the ontogeny of the clock support this notion, though there is also limited evidence that rhythmic gene expression can persist even in the absence of a functional molecular clock (64, 65). New evidence suggests that the appearance of robust rhythms of clock gene expression in mature follicles may be due to FSH-dependent expression of gap junction proteins (30). Disruption of cell-to-cell communication via gap junction blockers (e.g., lindane) reduces the amplitude and lengthens the period of PER2-luc expression in rat granulosa cells (30). These data indicate that gonadotropin-dependent communication among follicular cells may play a role in the appearance and/or maintenance of clock controlled gene (CCG) expression.

Gonadotropins clearly affect the timing and amplitude of clock gene expression in ovarian cells (20, 26, 27, 29, 63). We have systematically determined the phasic nature of sensitivity to gonadotropins in cultured rat granulosa cells (27). The physiological significance of these results is puzzling, given the transient nature of the follicle (29). Rather than mediating entrainment, it is likely that the impact of gonadotropins on the timing of clock gene expression reflects the indirect influence of receptor-mediated activation of cAMP-dependent signaling pathways (66).

Despite all the evidence for a molecular oscillator in follicular cells, the physiological significance of the ovarian clock is largely a mystery. Our own work reveals that the timing of ovulation may depend on a window of sensitivity to gonadotropins. We have observed a circadian rhythm of sensitivity to exogenous LH-treatment following suppression of endogenous LH secretion with a selective GnRHR antagonist (28). We have more recently determined that this rhythm is not dependent on the mature pattern of ovarian steroid hormone secretion or a fully developed and sexually mature neuroendocrine system, as we have observed the same rhythm in juvenile mice primed with equine gonadotropins (unpublished observation). These data indicate that rhythmic sensitivity of the ovary to gonadotropins may be an innate feature of the mature preovulatory follicle, driven in part by the ovarian clock.

How might the clock in follicular cells regulate the timing of sensitivity and/or prepare the preovulatory follicle for ovulation at the appropriate time? It is well known that the LH surge induces a significant change in gene expression within the granulosa and theca cells of the follicle (66–68). However, evidence for rhythmic expression of LH-responsive genes is limited. Several genes induced by LH signaling in the ovary are possible CCG candidates. LRH-1 (also known as CYP7A promoter binding factor) was first cloned and identified as an orphan nuclear receptor in the liver (69). In the ovary, LRH-1 expression is limited to the granulosa cell layer and is implicated in the regulation of steroid hormone biosynthesis and bile acid production (70). Recently, LRH-1 was shown to bind directly to CLOCK (71) and act synergistically to drive CLOCK:BMAL1 mediated transcription in the liver (71). In the ovary, LRH-1 has been implicated in the control of steroid biosynthesis in granulosa cells through direct activation of cytochrome P450 side chain cleavage (CYP11A1) transcription (72). Thus, LRH-1 may represent a mechanistic link between LH receptor signaling and the molecular clock in follicular cells.

In response to the LH surge enzymatic pathways responsible for follicular rupture are activated (67, 73, 74). A significant step in the response to LH is an increase in the level of prostanoids, including prostaglandin E2 (PGE2) and PGF2α (74). The rate-limiting enzyme for prostaglandin (PG) synthesis is cyclooxygenase-2 [COX2; (74)]. COX2 catalyzes the conversion of arachidonic acid to PGs and evidence suggests that COX2 expression is regulated by E-box promoter elements (74). In addition, treatment with PGE2 *in vivo* has been shown to phase shift the rhythm of *per1*, *d-element binding protein (dbp)*, and *rev-erb*α mRNA expression in the heart, liver, and kidney (75). Most recently, it was revealed that luteinized or “mature” granulosa cells do in fact have robust circadian rhythms of *ptgs2* and *lhcgr* gene expression that are disrupted and in some cases abolished by *bma11* siRNA (76). Together, these data suggest that an increase in COX2 and LH receptor expression and/or PG activity preceding the arrival of the LH surge may allow for predictive changes in ovarian cells in anticipation of ovulation.

It is also clear, from work in both rodents (30, 31, 36, 76) and birds (35), that the circadian clock plays a considerable role in the amplitude and timing of steroid hormone biosynthesis. Circadian rhythms of steroidogenic acute regulatory protein (StAR), 3beta-hydroxysteroid dehydrogenase (3β-HSD), 11α-hydroxylase, and aromatase (cyp19) have been observed in mature granulosa cells (30, 76). These rhythms are altered or abolished following treatment with *bmal1* siRNA (76). Further, *bmal1*^−/−^ mice have abnormally low levels of progesterone secretion due to reduced StAR expression (31).

## The Circadian Clock in the Uterus and Oviduct

Evidence for circadian clock function in the uterus is limited but supports a contribution of the uterine clock in the process of implantation, development of the conceptus, and eventual parturition (17, 23, 31, 33). Johnson and colleagues were the first to describe rhythmic clock gene expression in the uterus (17). Subsequent investigations determined that uterine cells were in fact semi-autonomous clocks (21, 32, 77). The timing of clock gene expression in the uterus appears to be affected by the reproductive cycle (78) and stimulation with ovarian steroids (79, 80). Global knockout of the core clock gene *bmal1* disrupts implantation, alters the level of steroid hormone synthesis, and compromises fertility (31). Further, targeted deletion of *bmal1* gene expression in the myometrium abrogates normal implantation (33). Finally, it was recently reported that circadian clock gene expression in endometrial stromal cells was attenuated during decidualization (81). In contrast with data from ovarian, testicular, and thymus tissue, where molecular clock function is linked to differentiation and maturation, these data suggest that silencing of the clock in uterine stromal cells may be permissive for cellular differentiation and maturation (81).

As with the uterus, initial evidence for a functional clock in the oviduct was provided nearly a decade ago by Johnson and colleagues (17). In the 10 years following few studies have advanced our understanding of clock function in this tissue. In fact, only one additional study by Kennaway and co-workers has examined clock function in the oviduct. These authors described rhythms of several clock genes and CCGs including *per2*, *bmal1*, *dbp*, *plasminogen activator inhibitor-1 (PAI-1)*, and *rev-erb* in the oviduct, supporting the notion that the embryo is exposed to rhythmic environmental conditions during passage to the uterus (18). Further the authors suggest that rhythmic secretory activity of epithelial cells may be critical for embryonic development. As with the ovary, additional functional studies of clock dependent physiology are needed to confirm the role of the clock in both the uterus and oviduct.

## Summary

The aim of this brief review is to discuss our current understanding of molecular clock function in the peripheral tissues of the mammalian female reproductive tract. It should be clear that, while we know a great deal about the location and character of the clock, our understanding of peripheral clock function is rather limited. The discovery of nearly ubiquitous clock gene expression in the tissues of the HPG axis suggests widespread and diverse physiological function. The female reproductive tract is fertile land for these explorations as it is elegantly organized, thoroughly integrated by positive and negative feedback, and temporally robust in its output. Using targeted deletion approaches (e.g., Cre-Lox system), investigators have begun to more thoroughly and intensively characterize molecular clock function in the uterus and pituitary gland. Extension of this approach to the ovary and oviduct will provide a more complete picture of clock function in these tissues.

It is clear that both adequate output of the SCN and coordination between the central pacemaker and peripheral clocks is critical for physiological homeostasis. Nowhere does this appear to be more true than in the reproductive axis. Rather than simply top–down hierarchical control, the HPG axis can be considered a partnership of synchronized and cooperative oscillators (Figure 1). The means of this synchronization remains to be adequately defined. Perhaps the system relies on the timing of adrenal glucocorticoids or autonomic nervous cues, each directly or indirectly influenced by the SCN [see Figure 1; (82, 83)]. More likely coordination of central and peripheral clocks is mediated by a synergy of multiple cues (82). Moreover, it is possible and even likely that feedback from these peripheral clocks acts to modulate the output of the central neuroendocrine network. This is certainly true of the HPG axis, as we have seen that ovarian steroids can affect central and peripheral oscillators, including the ovary itself. As the complex nature of the circadian timing system continues to be appreciated we are certain to discover how its function (and “dysfunction”) is an integral aspect of conditions that affect reproductive health and fertility.

## Conflict of Interest Statement

The author declares that the research was conducted in the absence of any commercial or financial relationships that could be construed as a potential conflict of interest.

## References

[1] Knobil E The neuroendocrine control of ovulation. Hum Reprod (1988) 3:469–72 3292570 10.1093/oxfordjournals.humrep.a136730

[2] Goldman BD Mahesh VB Porter JC The role of the ovary in control of cyclic LH release in the hamster, *Mesocricetus auratus*. Biol Reprod (1971) 4:57 5165063 10.1093/biolreprod/4.1.57

[3] Bronson FH Vom Saal FS Control of the preovulatory release of luteinizing hormone by steroids in the mouse. Endocrinology (1979) 104:1247–55 10.1210/endo-104-5-1247 571329

[4] Moenter SM Defazio AR Pitts GR Nunemaker CS Mechanisms underlying episodic gonadotropin-releasing hormone secretion. Front Neuroendocrinol (2003) 24:79–93 10.1016/S0091-3022(03)00013-X 12762999

[5] Everett JW Sawyer CH A 24-hour periodicity in the “LH-release apparatus” of female rats, disclosed by barbiturate sedation. Endocrinology (1950) 47:198 10.1210/endo-47-3-198 14793479

[6] Funabashi T Mitsushima D Nakamura TJ Uemura T Hirahara F Shinohara K Gonadotropin-releasing hormone (GnRH) surge generator in female rats. Prog Brain Res (2002) 141:165 10.1016/S0079-6123(02)41091-6 12508568

[7] Mitsushima D Tin Tin Win S Kimura F Sexual dimorphism in the GABAergic control of gonadotropin release in intact rats. Neurosci Res (2003) 46:399–405 10.1016/S0168-0102(03)00099-3 12871761

[8] Stetson MH Anderson PJ Circadian pacemaker times gonadotropin release in free-running female hamsters. Am J Physiol (1980) 238:R23–7 7188838 10.1152/ajpregu.1980.238.1.R23

[9] Mahoney MM Sisk C Ross HE Smale L Circadian regulation of gonadotropin-releasing hormone neurons and the preovulatory surge in luteinizing hormone in the diurnal rodent, *Arvicanthis niloticus*, and in a nocturnal rodent, *Rattus norvegicus*. Biol Reprod (2004) 70:1049–54 10.1095/biolreprod.103.021360 14668212

[10] Van der Beek EM Horvath TL Wiegant VM Van den HR Buijs RM Evidence for a direct neuronal pathway from the suprachiasmatic nucleus to the gonadotropin-releasing hormone system: combined tracing and light and electron microscopic immunocytochemical studies. J Comp Neurol (1997) 384:569 10.1002/(SICI)1096-9861(19970811)384:4<569::AID-CNE6>3.0.CO;2-0 9259490

[11] Van der Beek EM Wiegant VM Van Oudheusden HJ Van der Donk HA Van den HR Buijs RM Synaptic contacts between gonadotropin-releasing hormone-containing fibers and neurons in the suprachiasmatic nucleus and perichiasmatic area: an anatomical substrate for feedback regulation? Brain Res (1997) 755:101 10.1016/S0006-8993(97)00086-3 9163545

[12] Wiegand SJ Terasawa E Bridson WE Goy RW Effects of discrete lesions of preoptic and suprachiasmatic structures in the female rat. Alterations in the feedback regulation of gonadotropin secretion. Neuroendocrinology (1980) 31:147 10.1159/000123066 6771669

[13] Wiegand SJ Terasawa E Discrete lesions reveal functional heterogeneity of suprachiasmatic structures in regulation of gonadotropin secretion in the female rat. Neuroendocrinology (1982) 34:395–404 10.1159/000123335 6808412

[14] Sawyer CH Everett JW Markee JE A neural factor in the mechanism by which estrogen induces the release of luteinizing hormone in the rat. Endocrinology (1949) 44:218–33 10.1210/endo-44-3-218 18115073

[15] Albrecht U Timing to perfection: the biology of central and peripheral circadian clocks. Neuron (2012) 74:246–60 10.1016/j.neuron.2012.04.006 22542179

[16] Abe M Herzog ED Yamazaki S Straume M Tei H Sakaki Y Circadian rhythms in isolated brain regions. J Neurosci (2002) 22:350 11756518 10.1523/JNEUROSCI.22-01-00350.2002 PMC6757616

[17] Johnson MH Lim A Fernando D Day ML Circadian clockwork genes are expressed in the reproductive tract and conceptus of the early pregnant mouse. Reprod Biomed Online (2002) 4:140 10.1016/S1472-6483(10)61931-1 12470576

[18] Kennaway DJ Varcoe TJ Mau VJ Rhythmic expression of clock and clock-controlled genes in the rat oviduct. Mol Hum Reprod (2003) 9:503–7 10.1093/molehr/gag067 12900508

[19] Fahrenkrug J Georg B Hannibal J Hindersson P Gras S Diurnal rhythmicity of the clock genes Per1 and Per2 in the rat ovary. Endocrinology (2006) 147:3769–76 10.1210/en.2006-0305 16675517

[20] Karman BN Tischkau SA Circadian clock gene expression in the ovary: effects of luteinizing hormone. Biol Reprod (2006) 75:624–32 10.1095/biolreprod.106.050732 16807384

[21] He PJ Hirata M Yamauchi N Hashimoto S Hattori MA The disruption of circadian clockwork in differentiating cells from rat reproductive tissues as identified by in vitro real-time monitoring system. J Endocrinol (2007) 193:413–20 10.1677/JOE-07-0044 17535879

[22] Resuehr D Wildemann U Sikes H Olcese J E-box regulation of gonadotropin-releasing hormone (GnRH) receptor expression in immortalized gonadotrope cells. Mol Cell Endocrinol (2007) 278:36–43 10.1016/j.mce.2007.08.008 17928134

[23] Ratajczak CK Herzog ED Muglia LJ Clock gene expression in gravid uterus and extra-embryonic tissues during late gestation in the mouse. Reprod Fertil Dev (2010) 22:743–50 10.1071/RD09243 20450826 PMC3816753

[24] Sellix MT Menaker M Circadian clocks in mammalian reproductive physiology: effects of the “other” biological clock on fertility. Discov Med (2011) 11:273–81 21524381

[25] Williams WP III Kriegsfeld LJ Circadian control of neuroendocrine circuits regulating female reproductive function. Front Endocrinol (2012) 3:60 10.3389/fendo.2012.00060 22661968 PMC3356853

[26] He PJ Hirata M Yamauchi N Hashimoto S Hattori MA Gonadotropic regulation of circadian clockwork in rat granulosa cells. Mol Cell Biochem (2007) 302:111–8 10.1007/s11010-007-9432-7 17483911

[27] Yoshikawa T Sellix M Pezuk P Menaker M Timing of the ovarian circadian clock is regulated by gonadotrophins. Endocrinology (2009) 150:4338–47 10.1210/en.2008-1280 19520783 PMC2736075

[28] Sellix MT Yoshikawa T Menaker M A circadian egg timer gates ovulation. Curr Biol (2010) 20:R266–7 10.1016/j.cub.2010.01.045 20334830 PMC2888283

[29] Gras S Georg B Jorgensen HL Fahrenkrug J Expression of the clock genes Per1 and Bmal1 during follicle development in the rat ovary. Effects of gonadotropin stimulation and hypophysectomy. Cell Tissue Res (2012) 350:539–48 10.1007/s00441-012-1489-2 22940729

[30] Chen H Zhao L Chu G Kito G Yamauchi N Shigeyoshi Y FSH induces the development of circadian clockwork in rat granulosa cells via a gap junction protein Cx43-dependent pathway. Am J Physiol Endocrinol Metab (2013) 304:E566–75 10.1152/ajpendo.00432.2012 23299500

[31] Ratajczak CK Boehle KL Muglia LJ Impaired steroidogenesis and implantation failure in Bmal1-/- mice. Endocrinology (2009) 150:1879–85 10.1210/en.2008-1021 19056819 PMC5393263

[32] Akiyama S Ohta H Watanabe S Moriya T Hariu A Nakahata N The uterus sustains stable biological clock during pregnancy. Tohoku J Exp Med (2010) 221:287–98 10.1620/tjem.221.287 20647694

[33] Ratajczak CK Asada M Allen GC Mcmahon DG Muglia LM Smith D Generation of myometrium-specific Bmal1 knockout mice for parturition analysis. Reprod Fertil Dev (2012) 24:759–67 10.1071/RD11164 22697126

[34] Alvarez JD Sehgal A The thymus is similar to the testis in its pattern of circadian clock gene expression. J Biol Rhythms (2005) 20:111–21 10.1177/0748730404274078 15834108

[35] Nakao N Yasuo S Nishimura A Yamamura T Watanabe T Anraku T Circadian clock gene regulation of steroidogenic acute regulatory protein gene expression in preovulatory ovarian follicles. Endocrinology (2007) 148:3031–8 10.1210/en.2007-0044 17431006

[36] Alvarez JD Hansen A Ord T Bebas P Chappell PE Giebultowicz JM The circadian clock protein BMAL1 is necessary for fertility and proper testosterone production in mice. J Biol Rhythms (2008) 23:26–36 10.1177/0748730407311254 18258755 PMC2862364

[37] Kennaway DJ The role of circadian rhythmicity in reproduction. Hum Reprod Update (2005) 11:91 10.1093/humupd/dmh054 15569698

[38] Boden MJ Kennaway DJ Circadian rhythms and reproduction. Reproduction (2006) 132:379–92 10.1530/rep.1.00614 16940279

[39] Kovanen L Saarikoski ST Aromaa A Lonnqvist J Partonen T ARNTL (BMAL1) and NPAS2 gene variants contribute to fertility and seasonality. PLoS ONE (2010) 5:e10007 10.1371/journal.pone.0010007 20368993 PMC2848852

[40] Sellix MT Murphy ZC Menaker M Excess androgen during puberty disrupts circadian organization in female rats. Endocrinology (2013) 154:1636–47 10.1210/en.2012-2066 23417420 PMC3602624

[41] Goldman BD Kamberi IA Siiteri PK Porter JC Temporal relationship of progestin secretion, LH release and ovulation in rats. Endocrinology (1969) 85:1137 5388412 10.1210/endo-85-6-1137

[42] Gallo RV Neuroendocrine regulation of pulsatile luteinizing hormone release in the rat. Neuroendocrinology (1980) 30:122–31 10.1159/000122986 6101909

[43] Seibel MM Luteinizing hormone and ovulation timing. J Reprod Med (1986) 31:754–9 3531511

[44] Lewy H Naor Z Ashkenazi IE From ultradian to infradian rhythms: LH release patterns in vitro. Chronobiol Int (1999) 16:441–50 10.3109/07420529908998719 10442238

[45] Kerdelhue B Brown S Lenoir V Queenan JT Jr Jones GS Scholler R Timing of initiation of the preovulatory luteinizing hormone surge and its relationship with the circadian cortisol rhythm in the human. Neuroendocrinology (2002) 75: 158–63 10.1159/000048233 11914587

[46] Lewy H Naor Z Ashkenazi IE Rhythmicity of luteinizing hormone secretion expressed in vitro. Eur J Endocrinol (1996) 135:455 10.1530/eje.0.1350455 8921829

[47] Yamazaki S Numano R Abe M Hida A Takahashi R Ueda M Resetting central and peripheral circadian oscillators in transgenic rats. Science (2000) 288:682 10.1126/science.288.5466.682 10784453

[48] Shieh KR Distribution of the rhythm-related genes rPERIOD1, rPERIOD2, and rCLOCK, in the rat brain. Neuroscience (2003) 118:831 10.1016/S0306-4522(03)00004-6 12710990

[49] Yoo SH Yamazaki S Lowrey PL Shimomura K Ko CH Buhr ED PERIOD2::LUCIFERASE real-time reporting of circadian dynamics reveals persistent circadian oscillations in mouse peripheral tissues. Proc Natl Acad Sci U S A (2004) 101:5339 10.1073/pnas.0308709101 14963227 PMC397382

[50] Leclerc GM Boockfor FR Pulses of prolactin promoter activity depend on a noncanonical E-box that can bind the circadian proteins CLOCK and BMAL1. Endocrinology (2005) 146:2782–90 10.1210/en.2005-0100 15774559

[51] Resuehr HE Resuehr D Olcese J Induction of mPer1 expression by GnRH in pituitary gonadotrope cells involves EGR-1. Mol Cell Endocrinol (2009) 311:120–5 10.1016/j.mce.2009.07.005 19616057

[52] Chu A Zhu L Blum ID Mai O Leliavski A Fahrenkrug J Global but not gonadotrope-specific disruption of Bmal1 abolishes the luteinizing hormone surge without affecting ovulation. Endocrinology (2013). 10.1210/en.2013-1080 [Epub ahead of print]. 23736292

[53] Kakar SS Winters SJ Zacharias W Miller DM Flynn S Identification of distinct gene expression profiles associated with treatment of LbetaT2 cells with gonadotropin-releasing hormone agonist using microarray analysis. Gene (2003) 308:67–77 10.1016/S0378-1119(03)00446-3 12711391

[54] Olcese J Sikes HE Resuehr D Induction of PER1 mRNA expression in immortalized gonadotropes by gonadotropin-releasing hormone (GnRH): involvement of protein kinase C and MAP kinase signaling. Chronobiol Int (2006) 23:143–50 10.1080/07420520500521996 16687288

[55] Childs GV Unabia G Wu P Differential expression of growth hormone messenger ribonucleic acid by somatotropes and gonadotropes in male and cycling female rats. Endocrinology (2000) 141:1560–70 10.1210/en.141.4.1560 10746664

[56] Carbajo-Perez E Watanabe YG Cellular proliferation in the anterior pituitary of the rat during the postnatal period. Cell Tissue Res (1990) 261:333–8 10.1007/BF00318674 2401005

[57] Oishi Y Okuda M Takahashi H Fujii T Morii S Cellular proliferation in the anterior pituitary gland of normal adult rats: Influences of sex, estrous cycle, and circadian change. Anat Rec (1993) 235:111 10.1002/ar.1092350111 8417618

[58] Wunderer F Kuhne S Jilg A Ackermann K Sebesteny T Maronde E Clock gene expression in the human pituitary gland. Endocrinology (2013) 6:2046–57 10.1210/en.2012-2274 23584858

[59] Tischkau SA Jaeger CD Krager SL Circadian clock disruption in the mouse ovary in response to 2,3,7,8-tetrachlorodibenzo-p-dioxin. Toxicol Lett (2011) 201:116–22 10.1016/j.toxlet.2010.12.013 21182907 PMC3039055

[60] Nakao N Yasuo S Iigo M Ebihara S Yoshimura T Existence of ovulatory clock in the largest follicle on Japanese quail. 1st World Congress of Chronobiology. Sapporo (2004).

[61] Nakao N Yasuo S Ebihara S Yoshimura T Circadian expression of clock genes in cultured chicken granulosa cells. Ninth Meeting of the Society for Research on Biological Rhythms. Whistler, BC (2004).

[62] Chu G Yoshida K Narahara S Uchikawa M Kawamura M Yamauchi N Alterations of circadian clockworks during differentiation and apoptosis of rat ovarian cells. Chronobiol Int (2011) 28:477–87 10.3109/07420528.2011.589933 21797776

[63] Chu G Misawa I Chen H Yamauchi N Shigeyoshi Y Hashimoto S Contribution of FSH and triiodothyronine to the development of circadian clocks during granulosa cell maturation. Am J Physiol Endocrinol Metab (2012) 302:E645–53 10.1152/ajpendo.00470.2011 22205630

[64] Yamazaki S Yoshikawa T Biscoe EW Numano R Gallaspy LM Soulsby S Ontogeny of circadian organization in the rat. J Biol Rhythms (2009) 24:55–63 10.1177/0748730408328438 19150929 PMC2665126

[65] Li C Yu S Zhong X Wu J Li X Circadian rhythms of fetal liver transcription persist in the absence of canonical circadian clock gene expression rhythms in vivo. PLoS ONE (2012) 7:e30781 10.1371/journal.pone.0030781 22383974 PMC3285613

[66] Richards JS Ovulation: new factors that prepare the oocyte for fertilization. Mol Cell Endocrinol (2005) 234:75–9 10.1016/j.mce.2005.01.004 15836955

[67] Espey LL Richards JS Temporal and spatial patterns of ovarian gene transcription following an ovulatory dose of gonadotropin in the rat. Biol Reprod (2002) 67:1662–70 10.1095/biolreprod.102.005173 12444039

[68] Espey LL Ujioka T Okamura H Richards JS Metallothionein-1 messenger RNA transcription in steroid-secreting cells of the rat ovary during the periovulatory period. Biol Reprod (2003) 68:1895–902 10.1095/biolreprod.102.013557 12606366

[69] Nitta M Ku S Brown C Okamoto AY Shan B CPF: an orphan nuclear receptor that regulates liver-specific expression of the human cholesterol 7alpha-hydroxylase gene. Proc Natl Acad Sci U S A (1999) 96:6660–5 10.1073/pnas.96.12.6660 10359768 PMC21971

[70] Liu DL Liu WZ Li QL Wang HM Qian D Treuter E Expression and functional analysis of liver receptor homologue 1 as a potential steroidogenic factor in rat ovary. Biol Reprod (2003) 69:508–17 10.1095/biolreprod.102.011767 12672674

[71] Oiwa A Kakizawa T Miyamoto T Yamashita K Jiang W Takeda T Synergistic regulation of the mouse orphan nuclear receptor SHP gene promoter by CLOCK-BMAL1 and LRH-1. Biochem Biophys Res Commun (2007) 353:895–901 10.1016/j.bbrc.2006.12.131 17204240

[72] Kim JW Havelock JC Carr BR Attia GR The orphan nuclear receptor, liver receptor homolog-1, regulates cholesterol side-chain cleavage cytochrome p450 enzyme in human granulosa cells. J Clin Endocrinol Metab (2005) 90:1678–85 10.1210/jc.2004-0374 15613430

[73] Richards JS Russell DL Ochsner S Espey LL Ovulation: new dimensions and new regulators of the inflammatory-like response. Annu Rev Physiol (2002) 64:69–92 10.1146/annurev.physiol.64.081501.131029 11826264

[74] Sirois J Sayasith K Brown KA Stock AE Bouchard N Dore M Cyclooxygenase-2 and its role in ovulation: a 2004 account. Hum Reprod Update (2004) 10:373–85 10.1093/humupd/dmh032 15205395

[75] Tsuchiya Y Minami I Kadotani H Nishida E Resetting of peripheral circadian clock by prostaglandin E2. EMBO Rep (2005) 6:256–61 10.1038/sj.embor.7400356 15723041 PMC1299266

[76] Chen H Zhao L Kumazawa M Yamauchi N Shigeyoshi Y Hashimoto S Down-regulation of core clock gene Bmal1 attenuates expression of progesterone and prostaglandin biosynthesis-related genes in rat luteinizing granulosa cells. Am J Physiol Cell Physiol (2013) 12:C1131–40 10.1152/ajpcell.00008.2013 23596172

[77] Nakamura TJ Moriya T Inoue S Shimazoe T Watanabe S Ebihara S Estrogen differentially regulates expression of Per1 and Per2 genes between central and peripheral clocks and between reproductive and nonreproductive tissues in female rats. J Neurosci Res (2005) 82:622–30 10.1002/jnr.20677 16273538

[78] Nakamura TJ Sellix MT Kudo T Nakao N Yoshimura T Ebihara S Influence of the estrous cycle on clock gene expression in reproductive tissues: effects of fluctuating ovarian steroid hormone levels. Steroids (2010) 75:203–12 10.1016/j.steroids.2010.01.007 20096720 PMC2835461

[79] Hirata M He PJ Shibuya N Uchikawa M Yamauchi N Hashimoto S Progesterone, but not estradiol, synchronizes circadian oscillator in the uterus endometrial stromal cells. Mol Cell Biochem (2009) 1–2:31–8 10.1007/s11010-008-9981-4 19096762

[80] Nakamura TJ Sellix MT Menaker M Block GD Estrogen directly modulates circadian rhythms of PER2 expression in the uterus. Am J Physiol Endocrinol Metab (2008) 295:E1025–31 10.1152/ajpendo.90392.2008 18728223 PMC2584820

[81] Uchikawa M Kawamura M Yamauchi N Hattori MA Down-regulation of circadian clock gene period 2 in uterine endometrial stromal cells of pregnant rats during decidualization. Chronobiol Int (2011) 28:1–9 10.3109/07420528.2010.522289 21182399

[82] Buijs RM Scheer FA Kreier F Yi C Bos N Goncharuk VD Organization of circadian functions: interaction with the body. Prog Brain Res (2006) 153:341–60 10.1016/S0079-6123(06)53020-1 16876585

[83] Menaker M Murphy ZC Sellix MT Central control of peripheral circadian oscillators. Curr Opin Neurobiol (2013). 10.1016/j.conb.2013.03.003 [Epub ahead of print]. 23537900

